# Effects of manipulated food availability and seasonality on innate immune function in a passerine

**DOI:** 10.1111/1365-2656.13822

**Published:** 2022-10-31

**Authors:** Merijn M. G. Driessen, Maaike A. Versteegh, Yoran H. Gerritsma, B. Irene Tieleman, Ido R. Pen, Simon Verhulst

**Affiliations:** ^1^ University of Groningen Groningen the Netherlands

**Keywords:** foraging effort, innate immunity, manipulation of food availability, meso‐population, zebra finch

## Abstract

1. The innate immune system is essential for survival, yet many immune traits are highly variable between and within individuals. In recent years, attention has shifted to the role of environmental factors in modulating this variation. A key environmental factor is food availability, which plays a major role in shaping life histories, and may affect resource allocation to immune function through its effect on nutritional state.
2. We developed a technique to permanently increase foraging costs in seed‐eating birds, and leveraged this technique to study the effects of food availability on the innate immune system over a 3‐year period in 230 zebra finches housed in outdoor aviaries. The immune components we studied were haptoglobin, ovotransferrin, nitric oxide, natural antibodies through agglutination, complement‐mediated lysis, and killing capacity of *Escherichia coli* and *Candida albicans*, covering a broad spectrum of the innate immune system. We explored the effects of food availability in conjunction with other potentially important variables: season, age, sex and manipulated natal brood size.
3. Increased foraging costs affected multiple components of the immune system, albeit in a variable way. Nitric oxide and agglutination levels were lower under harsh foraging conditions, while *Escherichia coli* killing capacity was increased. Agglutination levels also varied seasonally, but only at low foraging costs. *C. albicans* killing capacity was lower in winter, and even more so for animals in harsh foraging conditions that were raised in large broods. Effects of food availability on ovotransferrin were also seasonal, and only apparent in males. Haptoglobin levels were independent of foraging costs and season.
4. Males had higher levels of immune function than females for three of the measured immune traits. Innate immune function was independent of age and manipulated natal brood size.
5. Our finding that food availability affects innate immune function suggests that fitness effects of food availability may at least partially be mediated by effects on the immune system. However, food availability effects on innate immunity varied in direction between traits, illustrating the complexity of the immune system and precluding conclusions on the level of disease resistance.

## Graphical abstract

This paper explores the effects of an ecologically relevant manipulation of foraging effort on a broad array of six innate immune measures, while also looking at effects of development, season and sex.

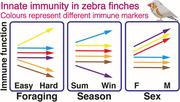

## 1 INTRODUCTION

The immune system provides a defence against pathogens and thereby benefits the health and survival of organisms (e.g. Roast et al., 2020). But despite its importance, immune function is a variable trait, showing significant differences between individuals, throughout the year and throughout life. Variation in traits is the outcome of an interplay of genetic and environmental components, but for the immune system, the variation that can be explained through additive genetic effects is often low (e.g. Ardia & Rice, 2006; Driessen et al., 2021 but see also Clapperton et al., 2009; Henryon et al., 2006), pointing to the environment as the dominant factor explaining immune variation. Environmental differences can indeed often be related to changes in various parts of the immune system. For example, geographical location and seasonal changes modulate immune system components (Buehler, Piersma, et al., 2008; Hegemann et al., 2012, 2013; Horrocks et al., 2015; Nwaogu et al., 2019; Versteegh et al., 2014). In recent years, attention has shifted to environmental conditions within the field of ecological immunology (Tieleman, 2018), but unravelling which aspects of the environment modulate the immune system, and how, has proven a complex puzzle. Many factors differ between environments at any particular time, and which factors are responsible for the differences in immune function is therefore largely unresolved. Candidate factors such as food availability/nutritional state, temperature, weather conditions and disease dynamics/risk of infection are known to differ between locations and seasons but are hard to separate from each other in a natural setting. We propose that experimental work in semi‐natural settings will help identify environmental effects on immune function.

Food availability is a likely candidate to explain variation in immune function between environments because survival and fecundity both vary with food availability (Boutin, 1990; Briga et al., 2017; Martin, 1987; Oro et al., 1999; Zanette et al., 2000). Maintenance of a well‐functioning immune system is thought to be costly (Hasselquist et al., 2001; Martin et al., 2003; Verhulst et al., 2005), consuming resources that could otherwise be used for other fitness‐enhancing processes, like reproduction (French, DeNardo, et al., 2007; French, Johnston, et al., 2007; Hanssen et al., 2004). Optimizing the distribution of available resources over these competing functions may shape variation in immune function and mediate life‐history trade‐offs (Sheldon & Verhulst, 1996). When energy is abundantly available, there is little need for compromise, but in general, trade‐offs will become stronger when food is harder to obtain. For example, a life‐history trade‐off between reproduction and survival in Great Tits *Parus major* was apparent in years followed by winters with poor foraging conditions, but not when foraging conditions were rich (Verhulst, 1998). Regarding the immune system, recent work on red crossbills *Loxia curvirostra* showed that immune parameters varied between years according to food abundance or scarcity (Schultz et al., 2020). This suggests that animals may compromise immune function when food is less readily available, and this effect will be stronger when the energy demands for other functions such as thermoregulation are higher.

Despite such predictions, effects of food availability on immune function are not well known. Due to interactions between food availability and other ecological factors, like predation risk and population density, it is difficult to identify the mechanisms underlying the effects of food availability on fitness components (Krebs et al., 1995; McNamara & Houston, 1987; Prevedello et al., 2013). Manipulating food availability in natural conditions does not fully resolve this, because predators, parasites and conspecifics may also respond to changes in food availability, mediated by changes in population density, thus confounding treatment effects. Furthermore, food availability is difficult to manipulate to a lower level in nature, so it is typically only increased. Food availability has been manipulated down frequently in laboratory studies through caloric restriction/dietary restriction (e.g. Nakagawa et al., 2012; Simons et al., 2013) and effects on physiology and life span have been extensively studied. However, such manipulations differ critically from variation in food availability under natural conditions, where food consumption is not constrained by the amount of food available but rather by the effort required to collect it (Wiersma et al., 2005). Natural food manipulations within semi‐natural settings are also scarce. Buehler et al. (2009) manipulated food availability through temporal restriction in red knots *Calidris canutus*, within a semi‐natural tidal flat setting where temporal food restrictions can come with the tides. They observed no effects of the food manipulation on immune function, although the sample size was small.

We here report the effects of foraging environment in adulthood on a broad array of innate immune measures in zebra finches (*Taeniopygia guttata*, Vieillot 1817) reared in either small or large broods (Driessen et al., 2021), in a full 2 × 2 design measured over multiple years and seasons. To manipulate the foraging environment, we applied a technique to permanently increase foraging costs (Koetsier & Verhulst, 2011), allowing us to estimate effects of manipulated food availability without confounding effects of predation and competition. We also investigated the effects of natal brood size manipulation and its interaction with the food availability manipulation. Previous work shows that growing up in a large brood did not affect innate immune function in adulthood within a benign foraging environment (Driessen et al., 2021), but the interaction with harsh foraging conditions was previously found to affect ageing and life span (Briga et al., 2017). These foraging and brood size manipulations are known to be effective in this model species (Briga et al., 2016, 2017; Jimeno et al., 2017; Koetsier & Verhulst, 2011), in which immune function is also relatively well studied (Deerenberg et al., 1997; Driessen et al., 2021; Kriengwatana et al., 2013; Naguib et al., 2004; Tschirren et al., 2009; Verhulst et al., 2005). One plausible outcome we predict is that, since harsh foraging and developmental conditions negatively affected life span, these manipulations will have a negative effect on overall immune function, thereby potentially contributing to the effect on life span. Likewise, given that females in our population have a shorter life span than males, we predicted lower immune function in females. We do not have specific predictions on how each of the separate immune components will be affected. In this respect, our study is strongly exploratory. Regarding season or seasonal differences in the effects of our manipulations we do not have clear predictions, and in this respect our study was also exploratory.

Animals were housed in outdoor aviaries, in groups that we refer to as meso‐populations. The semi‐natural conditions in these meso‐populations represent an ecologically relevant environment, while allowing for a level of experimental control not feasible in free‐living populations.

## 2 MATERIALS AND METHODS

### 2.1 Animals and treatment

In total, 230 Zebra finches were reared as described in Driessen et al. (2021). In brief, parental birds were mated randomly and were housed as pairs in cages (104 × 52 × 52 cm) with nesting material, cuttlefish bone, drinking water and commercial seed mixture provided ad libitum. Additional egg food supplementation was provided up to hatching. At the age of 1–5 days old, the chicks were randomly cross‐fostered to other nests to create small (2–3 chicks) and large (5–7 chicks) broods, forming our benign and harsh treatments, respectively. At 35 days old, the juveniles were moved to indoor aviaries (153 × 70 × 110 cm) with up to 30 same‐sex young and 2 adult pairs for sexual imprinting. Around 100 days old (range 90–120 days), individuals were moved to outdoor aviaries (310 × 210 × 150 cm), where they were housed with ad libitum food and water until they entered a foraging treatment (median: 316 days, range 158–780 days).

Foraging costs were manipulated as described by Koetsier and Verhulst (2011). In brief, each aviary has a food box attached to the ceiling, with five holes in each side through which food (tropical seed mixture) could be obtained. In the benign foraging environment (two aviaries), the food box had perches beneath the holes, while these were absent in the harsh foraging environment (also two aviaries), forcing birds to fly and hover to the holes in the food box to gather each individual seed. Experimental aviaries housed up to 24 individuals, with an approximately equal sex ratio.

All methods and experimental protocols were carried out under the approval of the Central Committee for Animal Experiments (Centrale Commissie Dierproeven) of The Netherlands, under licence AVD1050020174344. All methods were carried out in accordance with these approved guidelines.

Note that the immune data collected before the birds were exposed to either benign or harsh foraging conditions were previously published (Driessen et al., 2021) and serve as pre‐experimental baseline data with benign foraging conditions in the present study.

### 2.2 Blood sampling and processing

Birds were sampled biannually, in late winter (March) and late summer (September), from March 2018 to March 2020 (i.e. five sampling sessions), with the first sample for each bird taken before entering the foraging treatment. Within each sampling session, birds were sampled twice at a 2‐week interval.

To minimize potential handling stress effects (Buehler, Bhola, et al., 2008), no person had entered the aviary for at least 1 h prior to sampling, and we sampled birds within minutes after entering the aviary (median 4, range 1–10 min up to the end of sampling). Time taken for sampling was accounted for in the statistical analyses.

We sterilized the wing by cotton swab with 70% ethanol and collected blood (up to 150 μl) from the brachial vein in heparinized capillaries. Blood was immediately transferred into tubes on ice. Samples were centrifuged (10 min, 1500 g), and plasma was stored at −20°C for later analyses. The *C. albicans* killing assays were performed using fresh blood within an hour after sampling.

### 2.3 Immune tests

For all assays except the *C. albicans* killing assay, plasma of the two samples per individual, taken 2 weeks apart, was pooled to reduce stochastic variation and facilitate the distribution of plasma over different assays. Samples were randomly assigned to plates in all assays and all laboratory work was performed blind to bird identity and experimental treatments. The immune assays were performed in the following order, to minimize the potential effect of repeated freezing and thawing for certain assays (Hegemann et al., 2017; Liebl & Martin, 2009): (1) bacterial killing of *E. coli*, (2) haemolysis and hemagglutination, (3) haptoglobin, (4) nitric oxide and (5) ovotransferrin.

Samples had up to 5 freeze–thaw cycles before the last immune test and the number of cycles before an assay was constant because the assay sequence was fixed. All assays were performed within 2 months after the last blood sample was taken except for ovotransferrin, which was performed within 12 months after sampling. Previous experiments found no effect of storage for up to 6 years on ovotransferrin levels (Horrocks et al., 2011), and we therefore assumed the 12‐month storage time would have no effect.

All immune assays except for *C. albicans* and *E. coli* killing were performed as described in Driessen et al. (2021).

#### 2.3.1 Killing of *Candida albicans*

*C. albicans* killing capacity is a measure of constitutive innate immunity, showing how effectively the immune system combats a potential fungal infection using phagocytes. *C. albicans* killing capacity was tested in whole blood, as described by Millet et al. (2007).

In short, lyophilized pellets with *C. albicans* (Epower Assayed Microorganism, ATCC #10231; MicroBioLogics Inc.) were reconstituted in PBS, according to the manufacturer's instructions. From these stock solutions, we created fresh working solutions daily, which yielded about 200 colony‐forming units per 70 μl of medium‐blood‐microorganism mixture on our plates. Stock solutions were kept in the fridge for up to 3 days. We diluted 40 μl of whole blood in 160 μl CO_2_‐independent medium (Cat #18045088; Gibco‐Invitrogen), supplemented 5% FCS (Cat #10500056; ThermoFisher Scientific) and 4 mM l‐Glutamine (Cat #25030081; Gibco‐Invitrogen). The assay was started when 20 μl of *C. albicans* working solution was added to the diluted blood. The mixture was incubated at 41°C for 4 h, after which 70 μl was plated on tryptic soy agar plates in duplicate. To determine microbe growth and the number of micro‐organisms added at time zero, we made controls with 20 μl of *C. albicans* working solution in 200 μl medium, plated in duplicate. Inverted plates were incubated at 37°C for 48 h. After 2 days, the number of microbial colonies was counted and compared to controls. Experimental counts were divided by the control counts, to obtain a growth ratio. Duplicates had a high repeatability (*r* = 0.98). The score is expressed as $1-\frac{\text{sample}}{\text{Positive control}}$. Note that also in the case of average net growth (score <0) we refer to ‘killing capacity’, as this is customary in the field.Microbial growth measured is a result of both bacterial killing capacity and the blood's nutritional value, as will be the case in vivo. Due to this nature, ratios above 1 are not uncommon in this essay (e.g. Buehler, Piersma, et al., 2008).

#### 2.3.2 Bacterial killing of *Escherichia coli*

This assay provides a measure of how effective the immune system combats a potential bacterial infection using natural antibodies and the complement system. In brief, we used spectrophotometry to compare bacterial growth on 96‐well agar plates with and without exposure to the killing capacity of the plasma samples (Eikenaar & Hegemann, 2016; French & Neuman‐Lee, 2012). Before testing our samples, we tested different plasma volumes (5 and 7 μl) and bacterial solution concentrations (10^4^ and 10^5^) to optimize the assay. For the final assays, we mixed 7 μl of plasma with 5 μl of a 10^4^ *E. coli* solution (E power micro‐organisms; ACTT 8739) into each well, with additional agar instead of plasma for the positive controls.

Samples were plated in duplicate (*r* = 0.97). Plates were incubated at 37°C and were scanned 10 h after the onset of incubation, at 600 nm. The score is expressed as $1-\frac{\text{sample}}{\text{Positive control}}$. Note that also in the case of average net growth (score <0) we refer to ‘killing capacity’, as this is customary in the field.

#### 2.3.3 Haptoglobin (Hp)

Haptoglobin is an acute phase protein, and its concentrations can rapidly increase manifold in response to infection. Baseline concentrations reflect health status and physiological condition (Hõrak et al., 2002, 2003), and are also predictive of the response to infection. Concentrations were measured based on colorimetric analysis, through the use of a commercial kit also used by Matson et al. (2012), by following the manufacturer's instructions (Tridelta Development). We mixed 2.5 μl plasma with reagents (240 μl) in a 96‐well plate, after which absorbance was recorded at 630 nm.

#### 2.3.4 Nitric oxide (NOx)

Nitric oxide is a multifunctional signalling molecule, which aids in killing parasites, virus‐infected cells and tumour cells. Concentration was measured based on an assay created by Sild and Hõrak (2009). In brief, we used a colorimetric assay, measuring the concentration of both nitrate and nitrite in 10 μl of plasma. Samples were plated on 96‐well plates and colouration was measured at 540 nm.

#### 2.3.5 Haemolysis and hemagglutination

This assay measures complement system activation (lysis) and red blood cell agglutination mediated by natural antibodies. We measured haemolysis and hemagglutination following the original protocol by Matson et al. (2005). In short, we incubated Rabbit erythrocytes (15 μl 1% suspension; Envigo) in serially diluted plasma. Agglutination and lysis were scored as titres from assay plate images recorded, respectively, 20 and 90 min after incubation. Samples were all scored twice, from randomized images, by the same person, blind to sample ID and plate. The coefficients of variation within and between plates were 0.087 and 0.077, respectively.

#### 2.3.6 Ovotransferrin

Ovotransferrin is an avian‐specific acute‐phase protein with iron‐binding and further immunomodulary functions. By binding free iron, an essential nutrient for bacterial growth, it limits bacterial infection. Samples were measured using a colorimetric technique which estimates the amount of iron required to saturate all ovotransferrin in a 10 μl plasma sample (Horrocks et al., 2011). Samples were plated in duplicate (*r* = 0.96) on 96‐well plates and colouration was measured at 570 nm.

### 2.4 Statistical analysis

All analyses were conducted with R (v4.0.3; R Core Team, 2020) in the RStudio IDE (v1.2.5019; RStudio Team, 2020). We fitted Bayesian univariate response models with the brms package (v2.14.4; Bürkner, 2017, 2018), interfaced with the MCMC sampler of RStan (v2.21.2; Stan Development Team, 2020).

Besides our predictor variable of primary interest, foraging treatment, we included developmental treatment, sex, season, time in foraging treatment, age at start of foraging treatment and two measures of handling stress: handling time pre‐puncture and handling time post‐puncture (until sample completion). We allowed for up to four‐way interactions between treatments, sex and season. For time in treatment, we fitted separate spline smoothers (brms' default thin plate splines with 5 knots) for each combination of development and foraging treatment. The other predictors were entered as additive only (Table S1). For haptoglobin, we also included sample redness as predictor, as it can lead to bias in this colorimetric assay (Matson et al., 2012). The total number of parameters per model ranged from 24 to 26 (Table S1).

Since lysis was observed in only nine samples, we omitted lysis from statistical analysis. Owing to difficulties in obtaining enough blood and problems due to the COVID‐19 pandemic, the sample size for *C. albicans* killing and ovotransferrin were substantially smaller than sample sizes for the other response variables. Since a multivariate response analysis is limited to complete records (i.e. no missing data for any response variable), which would severely limit our sample size, we fitted univariate models only.

All response variables and continuous predictors were standardized (median = 0, SD = 1) to facilitate comparison of effect sizes between predictors and to increase efficiency of the MCMC sampler. For population‐level (‘fixed’) effects, we used ‘weakly informative’ (Lemoine, 2019) Gaussian priors (mean = 0, SD = 1). For group‐level (‘random’) effects, we used the default priors of brms (half‐*t* density with 3 degrees of freedom) for standard deviations. Models were run with normal or skewed normal families, chosen for the best fit (Table S1).

For each model, we ran three chains with 2000 discarded warmup iterations, followed by 3333 sampling iterations, thus yielding 9999 posterior samples per model. Proper mixing of chains was monitored with trace plots and convergence of chains by verifying that *Ȓ* values were close to 1.00. Model fits were evaluated by inspecting posterior predictive checks, using the pp_check() function of brms.

To test hypotheses regarding model parameters, we calculated the probability of direction (*p*_d_; Makowski, Ben‐Shachar, Chen, et al., 2019), which is the posterior probability that a quantity (parameter or derived parameter) is positive or negative, whichever is the most probable. In other words, the *p*_d_‐value equals the proportion of the posterior density that has the same sign as the median of the posterior density; this value can conceptually be regarded as a Bayesian analogue of the frequentist *p*‐value, where it reports the complement of p (i.e. 1 − p). *p*_d_‐values were calculated using the BayestestR package (Makowski, Ben‐Shachar, & Lüdecke, 2019). Following Makowski, Ben‐Shachar, Chen, et al. (2019), we consider *p*_d_‐values between 95% and 97% as providing ‘weak evidence’, a *p*_d_‐value between 97% and 99% as ‘moderately strong evidence’, and a value greater than 99% as ‘strong evidence’. Values of below 95% were considered as no discernible evidence/effects. Regarding effect sizes (*D*), we follow Cohen (1988) in considering a standardized effect size (*D*) around 0.2 as ‘small’, around 0.5 as ‘medium’ and around 0.8 as ‘large’. We presented main effects by averaging or marginalizing over other effects: Effect sizes and *p*_d_ for model parameters were calculated by setting continuous predictor variables at their median value and by averaging over levels of factors. The difference between the posterior fits for the levels of a factor was used to test a hypothesis. For all immune traits, higher values can be interpreted as a stronger immune system.

In addition to the main effects of the treatments, we tested the effects of time in foraging treatment and its interaction with the foraging and developmental treatments.

We furthermore calculated individual repeatability over the multiple sampling sessions according.

(1) $$r_{1}=\frac{V_{I}}{V_{I}+V_{R}}$$

Here *V*_I_ is the variance attributed to the individual and *V*_R_ the residual variance.

Age is a well‐known modulator of immune function (Peters et al., 2019), and we tested for effects of age using time in treatment as a proxy (median = 128 days, interquartile range = 0–326 days), to look at ageing effects from the moment of treatment onwards. Due to the added complexity, we made an inventory of the added value to the model by comparing leave‐one‐out (LOO) information criteria, commonly used in Bayesian statistics to determine model fit, of models with and without time in treatment, as well as models with time in treatment interactions. Time in treatment did not improve the LOO score for any immune parameter, either as main effect or in interactions, and we therefore chose to exclude this variable from the models we present.

Figures were made using the ggplot2 package (Wickham, 2016), and show both the 95% credible intervals and the posterior standard deviation, which is conceptually related to the frequentist concept of standard error. A complete list of packages used during data analysis and visualization is found in Table S2.

## 3 RESULTS

### 3.1 Foraging treatment

**FIGURE 1:**
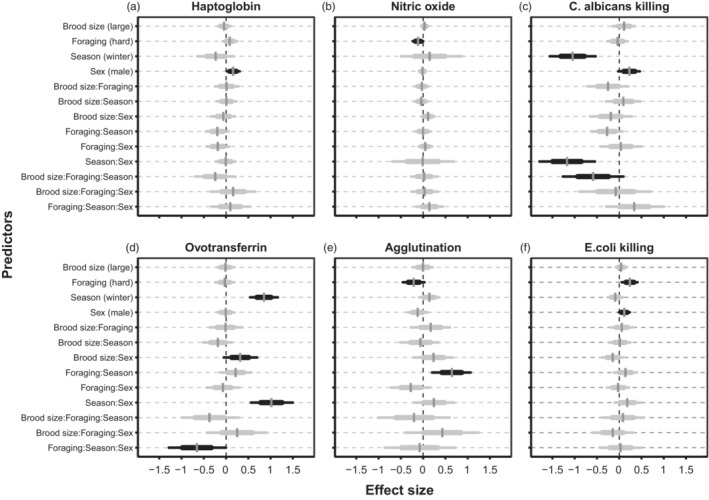
Estimated effect sizes for six immune components. The vertical bar shows the posterior mean, with thin bars showing 95% credible intervals, while thick bars show the posterior standard deviation. When probability of direction for an effect was greater than or equal to 0.95 (‘significant’), bars are coloured black. Effects are shown for the brood size manipulation (large–small), Foraging treatment (harsh–benign), Season (winter–summer), sex (male–female) and their interactions. The effect size of each predictor was calculated by setting other continuous predictor variables at their median value and by averaging over levels of other factors. Methodological variables that we control for, like age at start, handling time and sample redness, are not shown in the figure. Four‐way interactions were omitted from the figure, since there were no effects to be observed.

The foraging costs manipulation affected three out of six immune measures (Figure 1 and Figure S1). Birds living in harsh foraging conditions had lower nitric oxide levels (*D* = −0.11, *p*_d_ = 0.97; moderately strong evidence), higher *E. coli* killing capacity (*D* = 0.24, *p*_d_ = 1.00) and there was weak evidence for lower agglutination levels (*D =* −0.21, *p*_d_ = 0.96). Haptoglobin, killing capacity of *C. albicans* and ovotransferrin were not noticeably affected by the current foraging environment.

### 3.2 Natal brood size

Developmental conditions (small or large brood size) had no discernible effect on innate immune function, with all *p*_d_‐estimates well below 0.95 (Figure 1, Table S3), with the exception of an interaction between developmental treatment and conditions in adulthood for which there was weak evidence, with birds from large broods under harsh foraging conditions showed lower *C. albicans* killing capacity in winter (*D* = *−*0.59, *p*_d_ = 0.95). There was no discernible evidence for an interaction between developmental treatment and conditions in adulthood (foraging environment/season) with respect to the other five innate immune components we measured. There was weak evidence for an interaction with sex for ovotransferrin: males reared in large broods had higher ovotransferrin levels than males reared in small broods (*D* = 0.32, *p*_d_ = 0.95), while there was no such effect in females.

### 3.3 Season

**FIGURE 2:**
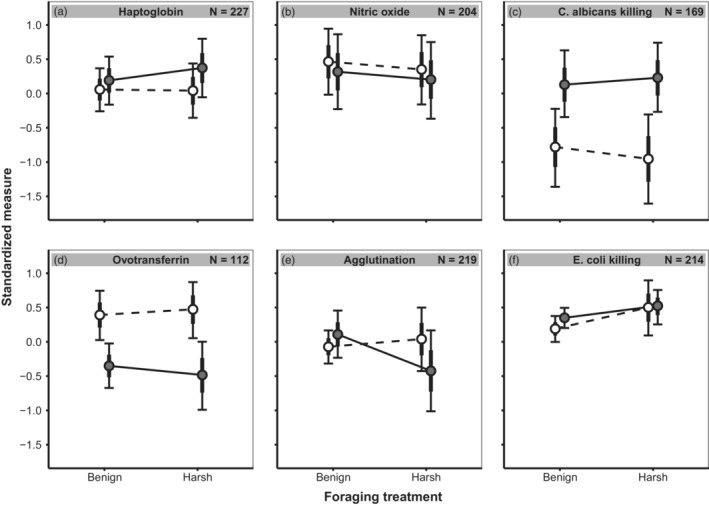
Standardized effects of foraging treatment and season on six immune indices. Shown are posterior means (circles), 95% credible intervals (thin bars) and posterior standard deviation (thick bars), during late winter (white, dashed line) and late summer (black, solid line). On the *y*‐axis are shown the standardized concentrations (a, b, d), titre (e), and killing capacities (c, f). Higher values indicate a stronger immune system.

Two out of six immune measures differed between seasons, with ovotransferrin (*D* = 0.85, *p*_d_ = 1.00) higher in late winter, and *C. albicans* killing capacity (*D* = *−*1.05, *p*_d_ = 1.00) lower in late winter. Furthermore, there was a season‐dependent effect of foraging treatment on agglutination, with birds in harsh foraging conditions having higher agglutination titres in late winter compared to late summer (*D* = 0.64, *p*_d_ = 1.00), while there was no such effect among birds in benign foraging conditions (Figure 2 and Figure S2). We also observed a season and sex‐dependent effect of foraging condition on ovotransferrin, with males having lower ovotransferrin concentrations in winter (*D* = *−*0.66, *p*_d_ = 0.98).

### 3.4 Sex

Male immune parameters were higher for 3 out 6 measures, while there was no sex effect on the other three measures (Figure 1). Haptoglobin levels (*D* = 0.16, *p*_d_ = 0.98) and *C. albicans killing capacity* (*D* = 0.23, *p*_d_ = 0.97) were higher in males, and there was also weak evidence for higher *E. coli* killing capacity (*D* = 0.11, *p*_d_ = 0.96). Furthermore, there was a sex‐specific effect of season, with large interaction effects for ovotransferrin (*D* = 1.02, *p*_d_ = 1.00) and *C. albicans* killing capacity (−1.18, *p*_d_ = 1.00). Males had higher ovotransferrin levels and lower *C. albicans* killing capacity than females in winter.

### 3.5 Repeatability

Individual repeatability measures between the different sampling rounds ranged from 0% to 20% (Table 1).

**TABLE 1:** Individual repeatability of innate immunity measures, calculated according to formula 1. Estimates are posterior means and 95% credible intervals.

	Haptoglobin	Nitric oxide	Agglutination	Ovotransferrin	*Candida albicans* killing	*Escherichia coli* killing
Mean	0.12	0.00	0.02	0.15	0.21	0.00
CI	0.03–0.22	0.00–0.02	0.00–0.10	0.01–0.31	0.08–0.35	0.00–0.02

## 4 DISCUSSION

Innate immune function is highly variable between and within individuals, and explaining this variation remains a major challenge. Heritability estimates of immune traits were low in the cohort we studied (see Driessen et al., 2021), suggesting dominance of environmental effects in causing immune trait variation, but which aspects of the environment drive this variation remains to be identified. To this end, we investigated the effects of foraging conditions experimentally, together with effects of season, sex and age in a semi‐natural environment that provided a compromise between ecological relevance and experimental control.

### 4.1 Foraging treatment

Effects of variation in food availability are different from starvation effects, because animals that experience prolonged differences in food availability can still remain in energy balance, as in the present study (Briga et al., 2019). However, an increase in foraging costs does generally result in a decrease in total energy consumption and re‐allocation of energy to foraging from other physiological functions, typically resulting in a decrease in basal metabolic rate (Wiersma & Verhulst, 2005) with concomitant effects on body temperature (e.g. Briga & Verhulst, 2017). We therefore expected the experimental increase in foraging costs to induce a reduction of immune function, as also often reported for reproductive effort manipulations (Knowles et al., 2009). In contrast, we find that effects of the foraging costs manipulation were complex: three out of six measures were affected, with nitric oxide and agglutination (weak evidence) levels decreased, and *E. coli* killing capacity increased under harsh foraging conditions. Thus, the main effect of lower food availability appears to be reallocation within the immune system rather than a general decline or improvement across the board. Whether such a reallocation of immune system investment is adaptive, or an unintended byproduct of having to work harder for food, is unclear at the moment, as is the overall effect of the reallocation on resistance against pathogens.

### 4.2 Developmental treatment

Using the same cohort of birds, it was previously shown that developmental hardship (growing up in a large brood) did not affect innate immune function in adulthood when exposed to a benign foraging environment (Driessen et al., 2021). However, developmental effects often depend on environmental quality experienced in adulthood (Costantini et al., 2014; Reid et al., 2003; Taborsky, 2006), also in our study population (Briga et al., 2017; Jimeno et al., 2017). We therefore anticipated potential effects of developmental conditions on innate immune function in a harsh foraging environment, despite the absence of such an effect in a benign foraging environment, but this prediction was not confirmed, with only weak evidence for an effect on *C. albicans* killing capacity. A potential explanation could be in the flexibility of the immune system, constantly adapting with the individual's current environment and internal state. Necessity to cater to this need could force individuals to overcome the observed short‐term effects of developmental hardship, potentially at the cost of other traits where we do see long‐term effects of developmental conditions (Jimeno et al., 2017, 2019; Montoya et al., 2018).

### 4.3 Season

With changing seasons, many environmental factors change. Decoupling these changes is important to further understand how the immune system is affected (Nwaogu et al., 2019). With our meso‐population setup, we were able to measure immune function in late winter and late summer in animals exposed to natural variation in photoperiod, temperature and humidity, while experiencing a constant food supply. This allows us to exclude food availability as a factor causing seasonal variation in immune function. Energy balance can however still be of effect, since seasonal fluctuations in ambient temperature causing variation in energy needs will modulate food availability effects.

Our results with respect to seasonal variation superficially resemble the effect of food availability, in that our findings were mixed, with main effects of season on two immune components, but in opposite directions: where ovotransferrin levels were higher in late winter*, C. albicans* killing capacity was lower, as indicated by higher growth. Additionally, we found an interaction between foraging treatment and season, with birds in harsh foraging conditions having lower levels of agglutination during late summer, and males having lower ovotransferrin concentrations during winter. It is interesting to note that the immune traits affected by foraging costs were mostly different from the traits that varied by season, showing that traits that might normally be seen as changing seasonally can specifically be attributed to changes in a specific factor like food availability, that changes with the seasons.

The mixed results for seasonal variation are not unexpected, and reflects the confluence of other factors that change seasonally. A potential factor to explanation the mixed results it that the optimal allocation to different immune system components varies seasonally according to seasonal shifts in actual or anticipated pathogen pressure. Since different pathogens are combatted by different parts of the immune system, it seems reasonable to suppose that the immune system can shift its focus in anticipation of seasonal changes in pathogen prevalence. There is evidence that environmental pathogen levels as well as bacterial infection levels and microbial loads vary seasonally (Horrocks et al., 2012; Nelson et al., 2002), so these seasonal changes in immune function may well be functional. Horrocks et al. found that seasonal changes innate immune parameters correlate with microbial loads on birds, but that microbial load on birds and within the environment followed different seasonal patterns. Measuring immune indices in conjunction with microbial load on the animals in different seasons could shed light on the functionality of observed seasonal changes in immune indices.

### 4.4 Sex‐specific effects

Sex differences in immunity are well described in mammals, in humans in particular (Klein & Flanagan, 2016), but for birds a general pattern has not yet been established (Hasselquist, 2007). A meta‐analysis of the effects of reproductive effort manipulations found little evidence for sex effects on either acquired immunity (immune responses against novel antigens) or parasitism (Knowles et al., 2009). However, Valdebenito et al. (2021) meta‐analysed acquired and innate immune measures, and found some immune parameters to be enhanced in males, but only during the breeding season. Most recently, Vincze et al. (2022) performed another meta‐analysis on birds during breeding season, where they found a female bias is most common. Our results add support to the existence of sex differences, looking at immune components most of which were not included within the meta‐analyses. We observed males to have higher scores in 3 out of 6 immune measures and no sex effect on the other three immune measures. In addition, we found sex‐specific effects to depend on season for ovotransferrin and *C. albicans* killing, where males differed more strongly between the seasons. In our study population, males consistently live longer than females (Briga et al., 2017), as appears to be a pattern in more birds (Clutton‐Brock & Isvaran, 2007). The sex difference in innate immunity may contribute to the life span difference (Metcalf et al., 2020), which could be verified by testing whether the sex‐dependent immune traits are predictors of life span.

### 4.5 Variability and repeatability

The assumed importance of the immune system for survival combined with the high variability and low individual repeatability we observed poses an apparent contradiction, because when changes in trait values have large fitness consequences those traits are expected to be strongly canalized, that is, show little variation (e.g. Boonekamp et al., 2018). However, the variation we observe at the immune trait level potentially reflects different resistance strategies, and ‘resistance’ at the level of the organism, that is, combining all aspects of immunity, may be less variable than variation at the level of the individual immune system traits.

## 5 CONCLUSIONS

Immune trait levels are highly dependent on the environment, and we studied the effects of two key environmental variables on innate immune function: food availability and season. Our study is novel in particular with respect to food availability, as we are not aware of earlier experiments that quantified effects of manipulated foraging costs on immune function. For both food availability and season, we found that the response differed between immune system components, in agreement with the finding that correlations between immune traits are generally low (Matson et al., 2006; Roast et al., 2019), also in our data (Driessen et al., 2021). Additionally, food availability and season affected different immune traits. Thus, at the level of individual traits our study leads to clear conclusions on the impact of food availability and season, but effects on the level of overall innate immune system functionality remain an open question. Given this complexity, how best to move forward? Identifying and quantifying the pathogens, or even all antigens, to which the immune system is responding may be essential, as well as developing insight in the effect of different immune trait‐level combinations on resistance against recent and current disease threats.

The immune system is complex, and, as confirmed in our study, immune traits do not change in unison. The relatively low individual repeatability also illustrates its variability, making it harder to observe manipulation effects or study longitudinal patterns. One way to increase our insight into effects on overall immune function, as well as functional defence against specific encountered pathogens, is the addition of in vivo immune challenges to test the functional effectiveness of the immune system. Usage of in vivo techniques in wild populations is tricky, seeing as one needs to capture animals multiple times per measurement, but is feasible in our semi‐natural setup. However, in vivo immune challenges can interfere with a longitudinal study design, since the initial challenge triggers the development of immunological memory, permanently affecting the immune system. For this reason, we abstained from such techniques in the present study. Recently, Peters et al. (2019) proposed ways to overcome the hurdles of in vivo immune testing in longitudinal study designs, and how options to potentially lower the number of sampling moments through a Planned Missed Data Design can further strengthen such an approach (Noble & Nakagawa, 2018).

## AUTHOR CONTRIBUTIONS

Merijn M. G. Driessen, B. Irene Tieleman, Ido R. Pen and Simon Verhulst conceived the ideas and designed the methodology; Merijn M. G. Driessen, Maaike A. Versteegh and Yoran H. Gerritsma collected the data; Merijn M. G. Driessen, Maaike A. Versteegh and Ido R. Pen analysed the data; Merijn M. G. Driessen and Simon Verhulst led the writing of the manuscript. All authors contributed critically to the drafts and gave final approval for publication.

## FUNDING INFORMATION

Merijn M. G. Driessen and Yoran H. Gerritsma were supported by Adaptive Life grants awarded by the University of Groningen.

## CONFLICT OF INTEREST

No competing interest to be declared.

## Supporting information


Appendix S1

## Data Availability

Data available from the Dryad Digital Repository https://doi.org/10.5061/dryad.jwstqjqct (Driessen et al., 2022).
