# Supplementary material for: Effects of manipulated food availability and seasonality on innate immune function in a passerine
Source: J Anim Ecol. 2022 Oct 31;91(12):2400–11. doi: 10.1111/1365-2656.13822 (PMC10092825; doi:10.1111/1365-2656.13822)
Supplement: Supplementary file 1 — Appendix S1 [file JANE-91-2400-s001.docx]

# Supplementary information


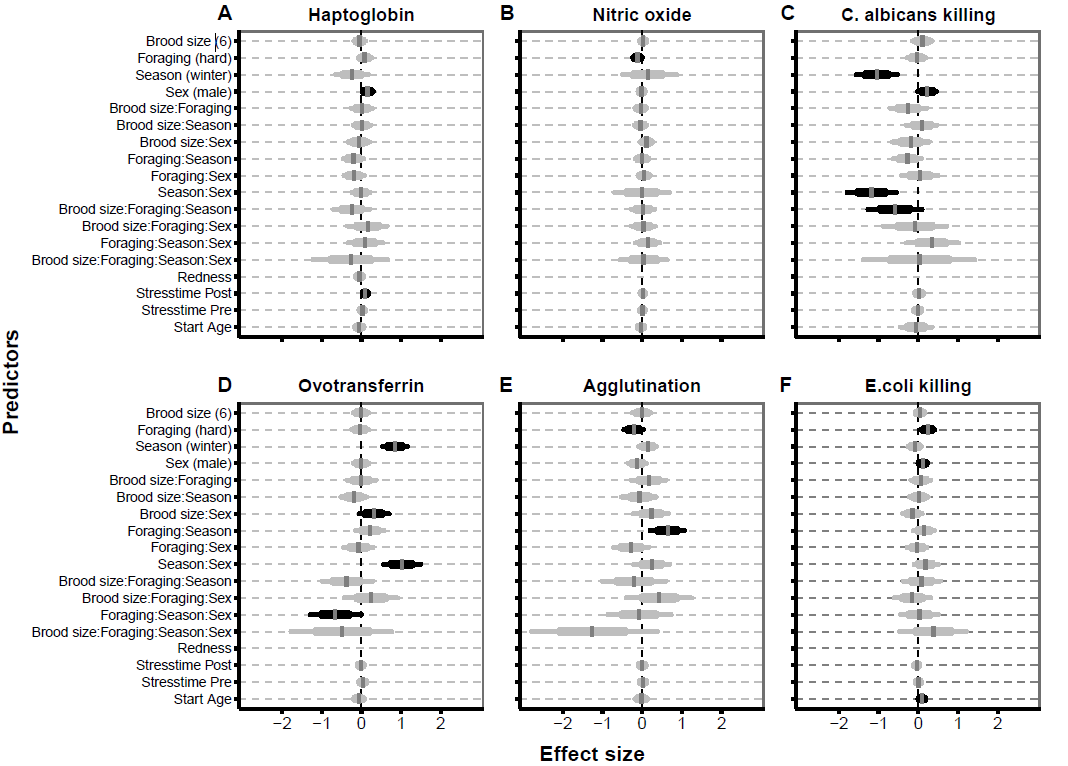


Figure S1: Estimated effect sizes for 6 immune components. The vertical bar shows the posterior mean, with thin bars showing 95% credible intervals, while thick bars show the posterior standard deviation. When probability of direction for an effect was greater than or equal to 0.95 ('significant'), bars are coloured black. Effects are shown for the brood size manipulation (large – small), Foraging treatment (harsh – benign), Season (winter – summer), sex (male – female) and their interactions. The effect size of each predictor was calculated by setting other continuous predictor variables at their median value and by averaging over levels of other factors.

Table S1: Model formulas (R code) for each of the response variables. Predictor variables are split into population-level (fixed) effects and group-level (random) effects.

| **Response variable** | **Population level effects formula** | **Group level effects formula** | **Number of parameters** | **Family** |
| --- | --- | --- | --- | --- |
| Haptoglobin | ~ Foraging * Brood size * Season * Sex + Age at start Foraging treatment + Handling time pre puncture + Handling time post puncture + Sample redness | (1\|BirdId) + (1\|Plate) | 26 | Skewed normal |
| Nitric oxide | ~ Foraging * Brood size * Season * Sex + Age at start Foraging treatment + Handling time pre puncture + Handling time post puncture | (1\|BirdId) + (1\|Plate) | 25 | Skewed normal |
| Agglutination | ~ Foraging * Brood size * Season * Sex + Age at start Foraging treatment + Handling time pre puncture + Handling time post puncture | (1\|BirdId) | 23 | Normal |
| Ovotransferrin | ~ Foraging * Brood size * Season * Sex + Age at start Foraging treatment + Handling time pre puncture + Handling time post puncture | (1\|BirdId) + (1\|Plate) | 25 | Normal |
| *C. albicans* killing | ~ Foraging * Brood size * Season * Sex + Age at start Foraging treatment + Handling time pre puncture + Handling time post puncture | (1\|BirdId) + (1\|Day) | 24 | Normal |
| *E. coli* killing | ~ Foraging * Brood size * Season * Sex + Age at start Foraging treatment + Handling time pre puncture + Handling time post puncture | (1\|BirdId) + (1\|Plate) | 25 | Skewed normal |

Table S2: A list of used packages in R during data analysis and visualization.

| **Package** | **Version** | **Reference** |
| --- | --- | --- |
| Bayesplot | 1.7.2 | (Gabry and Mahr, 2020) |
| BayestestR | 0.7.5 | (Makowski, Ben-Shachar and Lüdecke, 2019) |
| Brms | 2.14.4 | (Bürkner, 2017, 2018) |
| Dplyr | 1.0.2 | (Wickham *et al.*, 2020) |
| ggplot2 | 3.3.2 | (Wickham, 2016) |
| ggpubr | 0.4.0 | (Kassambara, 2018) |
| ggthemes | 4.2.0 | (Arnold, 2019) |
| Ggmap | 3.0.0 | (Kahle and Wickham, 2013) |
| loo | 2.3.1 | (Vehtari *et al.*, 2019) |
| Officer | 0.3.15 | (Gohel, 2019) |
| RODBC | 1.3.17 | (Ripley and Lapsley, 2017) |
| Rstan | 2.21.2 | (Stan Development Team, 2020) |
| Tidybayes | 2.3.1 | (Kay, 2020) |


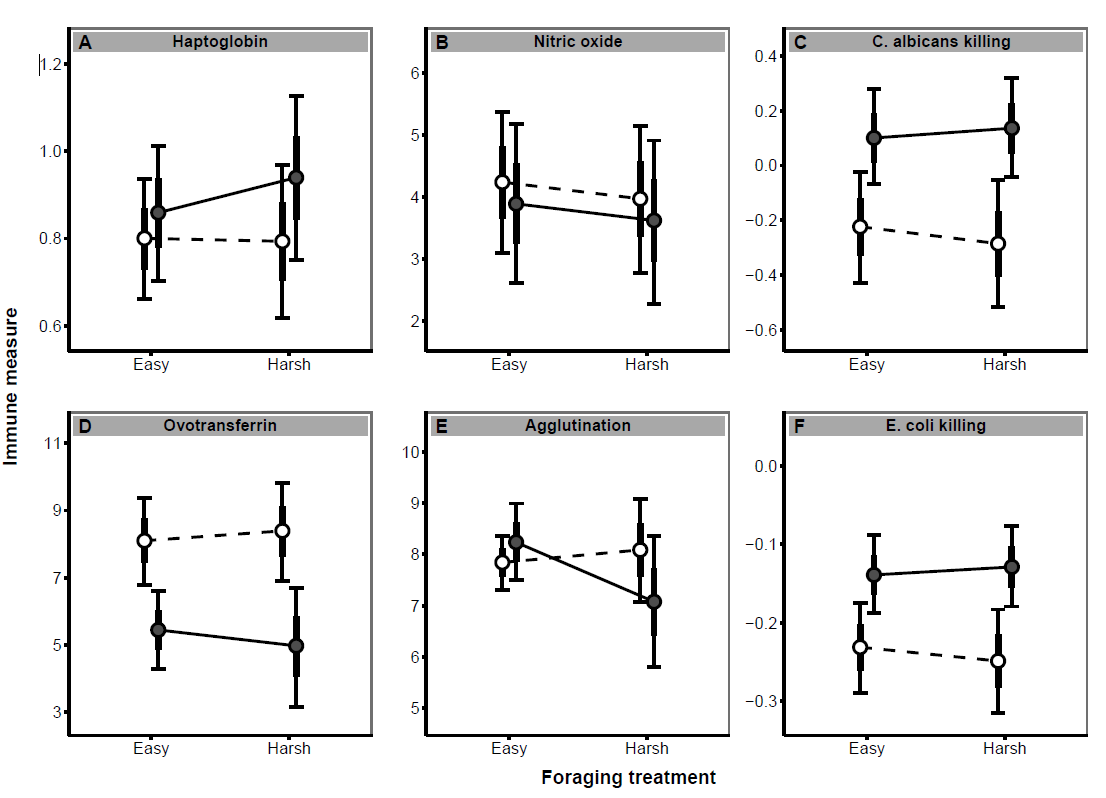


Figure S2: Effects of foraging treatment and season on six immune indices, with model estimates transformed back to original scale. Shown are posterior means (circles), 95% credible intervals (thin bars) and posterior standard deviation (thick bars), during late winter (white, dashed line) and late summer (black, solid line). On the y-axis are shown the concentrations (A,B,D), titre (E), and killing capacities (C,F).

|  | **Haptoglobin** | | **Nitric Oxide** | | **Agglutination** | | **Ovotransferrin** | | ***C. Albicans* killing** | | ***E. Coli* killing** | |
| --- | --- | --- | --- | --- | --- | --- | --- | --- | --- | --- | --- | --- |
|  | **Effect size**  **(95% CI)** | ***p*_d_** | **Effect size**  **(95% CI)** | ***p*_d_** | **Effect size**  **(95% CI)** | ***p*_d_** | **Effect size**  **(95% CI)** | ***p*_d_** | **Effect size**  **(95% CI)** | ***p*_d_** | **Effect size**  **(95% CI)** | ***p*_d_** |
| **Brood size (large)** | -0.05  (-0.19, 0.10) | 0.74 | 0.03  (-0.05, 0.11) | 0.78 | 0.00  (-0.24, 0.22) | 0.51 | -0.01  (-0.20, 0.19) | 0.55 | 0.11  (-0.15, 0.35) | 0.80 | 0.04  (-0.08, 0.17) | 0.74 |
| **Foraging (hard)** | 0.08  (-0.08, 0.25) | 0.83 | -0.11  (-0.24, 0.00) | 0.97ᶧ | -0.21  (-0.46, 0.03) | 0.96ᶧ | -0.03  (-0.24, 0.18) | 0.60 | -0.04  (-0.27, 0.19) | 0.62 | 0.24  (0.06, 0.41) | 1.00* |
| **Season (winter)** | -0.23  (-0.65, 0.18) | 0.88 | 0.15  (-0.49, 0.88) | 0.67 | 0.14  (-0.09, 0.36) | 0.89 | 0.85  (0.54, 1.16) | 1.00* | -1.05  (-1.56, -0.53) | 1.00* | -0.09  (-0.26, 0.08) | 0.85 |
| **Sex (male)** | 0.16  (0.02, 0.31) | 0.98* | -0.01  (-0.09, 0.06) | 0.63 | -0.12  (-0.35, 0.11) | 0.86 | -0.01  (-0.20, 0.19) | 0.53 | 0.23  (-0.02, 0.46) | 0.97ᶧ | 0.11  (-0.01, 0.24) | 0.96ᶧ |
| **Brood size : Foraging** | 0.01  (-0.25, 0.28) | 0.53 | -0.03  (-0.18, 0.12) | 0.65 | 0.17  (-0.28, 0.60) | 0.77 | -0.01  (-0.36, 0.37) | 0.52 | -0.25  (-0.71, 0.21) | 0.86 | 0.06  (-0.19, 0.31) | 0.69 |
| **Brood size : Season** | 0.01  (-0.21, 0.24) | 0.53 | -0.04  (-0.19, 0.11) | 0.70 | -0.06  (-0.51, 0.35) | 0.61 | -0.19  (-0.52, 0.13) | 0.87 | 0.09  (-0.30, 0.48) | 0.68 | 0.01  (-0.23, 0.25) | 0.55 |
| **Brood size : Sex** | -0.06  (-0.34, 0.20) | 0.67 | 0.11  (-0.04, 0.26) | 0.93 | 0.23  (-0.22, 0.66) | 0.85 | 0.32  (-0.05, 0.70) | 0.95ᶧ | -0.19  (-0.66, 0.28) | 0.79 | -0.15  (-0.39, 0.10) | 0.88 |
| **Foraging : Season** | -0.20  (-0.45, 0.05) | 0.94 | 0.00  (-0.18, 0.18) | 0.50 | 0.64  (0.20, 1.07) | 1.00* | 0.21  (-0.14, 0.57) | 0.89 | -0.27  (-0.63, 0.07) | 0.93 | 0.14  (-0.12, 0.40) | 0.85 |
| **Foraging : Sex** | -0.18  (-0.44, 0.07) | 0.93 | 0.05  (-0.10, 0.20) | 0.72 | -0.28  (-0.72, 0.17) | 0.89 | -0.07  (-0.44, 0.30) | 0.65 | 0.03  (-0.42, 0.5) | 0.56 | -0.03  (-0.28, 0.22) | 0.58 |
| **Season : Sex** | 0.00  (-0.23, 0.22) | 0.52 | -0.01  (-0.70, 0.69) | 0.51 | 0.24  (-0.21, 0.70) | 0.85 | 1.02  (0.56, 1.50) | 1.00* | -1.18  (-1.79, -0.54) | 1.00* | 0.18  (-0.11, 0.50) | 0.88 |
| **Brood size : Foraging : Season** | -0.24  (-0.70, 0.22) | 0.85 | 0.02  (-0.27, 0.32) | 0.55 | -0.20  (-1.01, 0.59) | 0.69 | -0.38  (-0.99, 0.29) | 0.88 | -0.59  (-1.27, 0.09) | 0.95* | 0.08  (-0.38, 0.56) | 0.64 |
| **Brood size : Foraging :**  **Sex** | 0.16  (-0.35, 0.65) | 0.73 | 0.04  (-0.26, 0.33) | 0.60 | 0.43  (-0.40, 1.24) | 0.85 | 0.25  (-0.43, 0.91) | 0.77 | -0.08  (-0.89, 0.71) | 0.57 | -0.15  (-0.60, 0.31) | 0.73 |
| **Foraging : Season :**  **Sex** | 0.09  (-0.34, 0.54) | 0.66 | 0.14  (-0.16, 0.45) | 0.82 | -0.08  (-0.86, 0.73) | 0.58 | -0.66  (-1.28, 0.00) | 0.98* | 0.34  (-0.29, 1.01) | 0.85 | 0.03  (-0.45, 0.50) | 0.55 |
| **Brood size : Foraging :**  **Season : Sex** | -0.27  (-1.22, 0.66) | 0.71 | 0.04  (-0.54, 0.62) | 0.55 | -1.26  (-2.78, 0.39) | 0.94 | -0.49  (-1.77, 0.77) | 0.77 | 0.03 (-1.38, 1.42 | 0.52 | 0.39 (-0.47, 1.21) | 0.81 |
| **Age at start of treatment** | -0.05  (-0.18, 0.07) | 0.79 | -0.03  (-0.11, 0.05) | 0.77 | -0.02  (-0.18, 0.14) | 0.59 | -0.07  (-0.21, 0.08) | 0.82 | -0.05  (-0.45, 0.35) | 0.61 | 0.09  (0.00, 0.18) | 0.98* |
| **Handling time pre puncture** | 0.03  (-0.03, 0.08) | 0.84 | 0.01  (-0.03, 0.05) | 0.66 | -0.02  (-0.07, 0.11) | 0.69 | 0.03  (-0.06, 0.13) | 0.75 | -0.02  (-0.12, 0.09) | 0.61 | 0.01  (-0.05, 0.05) | 0.58 |
| **Handling time post puncture** | 0.10  (0.05, 0.15) | 1.00* | 0.02  (-0.02, 0.05) | 0.83 | 0.00  (-0.09, 0.10) | 0.52 | -0.01  (-0.09, 0.06) | 0.65 | 0.02  (-0.09, 0.13) | 0.64 | -0.04  (-0.10, 0.01) | 0.93 |
| **Redness** | -0.05  (-0.15, 0.06) | 0.83 | - | - | - | - | - | - | - | - | - | - |

| Brood size | Foraging treatment | Season | Sex | **Haptoglobin** | **Nitric Oxide** | **Agglutination** | **Ovotransferrin** | ***C. Albicans* killing** | ***E. Coli* killing** |
| --- | --- | --- | --- | --- | --- | --- | --- | --- | --- |
| Benign | Benign | Winter | Female | 64 | 55 | 63 | 16 | 13 | 65 |
|  |  |  | Male | 61 | 53 | 59 | 22 | 10 | 61 |
|  |  | Summer | Female | 31 | 31 | 28 | 13 | 11 | 20 |
|  |  |  | Male | 39 | 39 | 37 | 23 | 18 | 27 |
|  | Harsh | Winter | Female | 19 | 19 | 19 | 10 | 7 | 17 |
|  |  |  | Male | 19 | 20 | 17 | 11 | 9 | 19 |
|  |  | Summer | Female | 17 | 17 | 16 | 7 | 15 | 13 |
|  |  |  | Male | 15 | 15 | 13 | 6 | 13 | 13 |
| Harsh | Benign | Winter | Female | 49 | 43 | 46 | 14 | 9 | 47 |
|  |  |  | Male | 50 | 43 | 50 | 17 | 6 | 51 |
|  |  | Summer | Female | 36 | 36 | 34 | 15 | 12 | 25 |
|  |  |  | Male | 33 | 33 | 28 | 19 | 9 | 22 |
|  | Harsh | Winter | Female | 20 | 21 | 21 | 13 | 6 | 19 |
|  |  |  | Male | 20 | 18 | 19 | 11 | 8 | 16 |
|  |  | Summer | Female | 13 | 13 | 12 | 5 | 11 | 11 |
|  |  |  | Male | 15 | 15 | 13 | 3 | 12 | 11 |

Table S3: The marginal effects with credible intervals of developmental treatment and interactions with age and sex, while keeping other predictors at their sample median (continuous predictors) or averaged over levels (factors). p_d_ values ≥ 0.95 are marked with an ᶧ and values ≥ 0.97 are marked with an * (based on unrounded values)

*Table S4: Sample sizes of subgroups for all immune measures*

# References

Arnold, J. B. (2019) ‘ggthemes: Extra Themes, Scales and Geoms for “ggplot2”’. Available at: https://cran.r-project.org/package=ggthemes.

Bürkner, P. C. (2017) ‘brms: An R package for Bayesian multilevel models using Stan’, *Journal of Statistical Software*, 80(1). doi: 10.18637/jss.v080.i01.

Bürkner, P. C. (2018) ‘Advanced Bayesian multilevel modeling with the R package brms’, *R Journal*, 10(1), pp. 395–411. doi: 10.32614/rj-2018-017.

Gabry, J. and Mahr, T. (2020) ‘bayesplot: Plotting for Bayesian Models’. Available at: https://mc-stan.org/bayesplot.

Gohel, D. (2019) ‘officer: Manipulation of Microsoft Word and PowerPoint Documents’. Available at: https://cran.r-project.org/package=officer.

Kahle, D. and Wickham, H. (2013) ‘ggmap: Spatial visualization with ggplot2’, *R Journal*, 5(1), pp. 144–161. doi: 10.32614/rj-2013-014.

Kassambara, A. (2018) ‘ggpubr: “ggplot2” Based Publication Ready Plots’. Available at: https://cran.r-project.org/package=ggpubr.

Kay, M. (2020) ‘tidybayes: Tidy Data and Geoms for Bayesian Models’. Available at: http://mjskay.github.io/tidybayes.

Makowski, D., Ben-Shachar, M. and Lüdecke, D. (2019) ‘bayestestR: Describing Effects and their Uncertainty, Existence and Significance within the Bayesian Framework’, *Journal of Open Source Software*, 4(40), p. 1541. doi: 10.21105/joss.01541.

Ripley, B. and Lapsley, M. (2017) ‘RODBC: ODBC Database Access’. Available at: https://cran.r-project.org/package=RODBC.

Stan Development Team (2020) ‘RStan: the R interface to Stan.’ Available at: http://mc-stan.org/.

Vehtari, A. *et al.* (2019) ‘loo: Efficient leave-one-out cross-validation and WAIC for Bayesian models’. Available at: https://cran.r-project.org/package=loo.

Wickham, H. (2016) *ggplot2: Elegant Graphics for Data Analysis.* New York: Springer-Verlag.

Wickham, H. *et al.* (2020) ‘dplyr: A Grammar of Data Manipulation.’
